# Diversity and abundance of macro‐invertebrates on abandoned cattle kraals in a semi‐arid savanna

**DOI:** 10.1002/ece3.4332

**Published:** 2018-07-30

**Authors:** Justice Muvengwi, Gift Chikorowondo, Monicah Mbiba, Edson Gandiwa

**Affiliations:** ^1^ Department of Natural Resources Bindura University of Science Education Bindura Zimbabwe; ^2^ Restoration and Conservation Biology Research Group School of Animal, Plant and Environmental Sciences University of the Witwatersrand Johannesburg South Africa; ^3^ School of Wildlife Ecology, and Conservation Chinhoyi University of Technology Chinhoyi Zimbabwe

**Keywords:** abandoned kraals, macro‐invertebrates, nutrient heterogeneity, nutrient hotspots, diversity

## Abstract

Abandoned cattle (*Bos taurus*) kraals are sources of habitat heterogeneity in dystrophic semi‐arid African savannas with a strong positive effect on soil nutrients and plant productivity. However, little is known regarding how macro‐invertebrate assemblages vary between abandoned kraals and the surrounding savanna matrix. We tested whether herbaceous biomass and basal and aerial covers and soil nutrients have an effect on aboveground and belowground macro‐invertebrate assemblages. Twelve abandoned kraals were contrasted with their paired control plots for soil characteristics, herbaceous productivity, and macro‐invertebrate assemblages in Save Valley Conservancy, Zimbabwe. Abandoned kraals had significantly higher concentrations of soil nitrogen (N), phosphorus (P), potassium (K), and calcium (Ca) as well as herbaceous biomass and basal and aerial covers than control plots. Both aboveground and belowground macro‐invertebrate species richness were higher on abandoned kraals. However, only belowground macro‐invertebrate diversity (Shannon *H′* and Hill number 1) was significantly higher on abandoned kraals. Soil nutrients and herbaceous productivity had positive and significant correlations with the dominant taxa (Coleoptera, Hymenoptera, Hemiptera, Isoptera, and Myriapoda) on abandoned kraals. These results add to the growing body of evidence that abandoned kraals exert significant effects on savanna spatial heterogeneity years later, with implications on ecosystem processes and functioning.

## 1 INTRODUCTION

The traditional extensive livestock husbandry practice in sub‐Saharan Africa involves the use of kraals (mostly made of thorn fence) as part of daily management (Augustine, 2003). Under this practice, livestock are allowed to graze in surrounding areas far from the kraals and are brought to the kraals overnight as a way of protecting them from theft and livestock predators among other reasons (Augustine, 2003; Blackmore, Mentis, & Scholes, 1990; Kizza, Totolo, Perkins, & Areola, 2010). The dung and urine dropped in the kraals result in the concentration of nutrients, which after kraal abandonment creates a mosaic of soil nutrient‐rich patches in otherwise dystrophic savanna landscapes (Augustine, 2003; Reid & Ellis, 1995).

Some of the soil nutrients that have been found to be higher on abandoned kraals compared with the surrounding savanna matrix across studies include nitrogen (N), phosphorus (P), potassium (K), calcium (Ca), and organic carbon (C) (Augustine, 2003; Chikorowondo, Muvengwi, Mbiba, & Gandiwa, 2018; Kizza et al., 2010; Porensky & Veblen, 2015; Young, Patridge, & Macrae, 1995). Soil nutrient‐rich sites have cascading effects on plant productivity, biodiversity, and preferential selection by wildlife (Augustine, 2003; Blackmore et al., 1990; Chikorowondo et al., 2018; Davies et al., 2014; Kizza, 2011; Muvengwi, Witkowski, Parrini, & Davies, 2017). The foraging herbivores attracted by the nutrient‐rich sites in turn create a positive feedback loop as they drop their dung and urine during foraging, further enriching these sites (Augustine, Mcnaughton, & Frank, 2003; Chikorowondo et al., 2018; van der Waal et al., 2011). Due to the dystrophic nature of African savannas, the effect of abandoned cattle kraals as nutrient‐rich patches is expected to have more profound effects on savanna ecosystem functioning.

Three to four decades ago, southern African savannas experienced land‐use change from livestock ranching to wildlife conservation (Lindsey, du Toit, Pole, & Romañach, 2008; van der Waal et al., 2011), because livestock ranching became uneconomical (Lindsey et al., 2008). This left a mosaic of abandoned cattle kraal sites that can be viewed as disturbed nutrient‐rich patches in the landscape (Chikorowondo et al., 2018; van der Waal et al., 2011). Disturbance of natural ecosystems may lead to the decline of aboveground plant biomass and diversity as well as the belowground macro‐invertebrates diversity (Hooper et al., 2000). Disturbance emanating from land‐use change decreased the diversity of both belowground and aboveground macro‐invertebrates, for example, termites (Eggleton et al., 2002; Muvengwi, Mbiba, Ndagurwa, Nyamadzawo, & Nhokovedzo, 2017), ants (Bestelmeyer & Wiens, 1996; Mauda, Joseph, Seymour, Munyai, & Foord, 2018), beetles (da Silva, Aguiar, Niemelä, Sousa, & Serrano, 2008; Nestel, Dickschen, & Altiere, 1993; Perfecto, Armbrecht, Philpott, Soto‐Pinto, & Dietsch, 2007), and nematodes (Freckman & Ettema, 1993; Liang, Lavian, & Steinberger, 2001). However, little is known on the effects of disturbance in the form of abandoned kraal sites on macro‐invertebrates diversity.

Abandoned kraals harbor unique plant species (Augustine, 2003; Young et al., 1995), and grass biomass production and cover have been found to be higher on abandoned kraals than the surrounding savanna (Chikorowondo et al., 2018; Porensky & Veblen, 2015; van der Waal et al., 2011). Plants are examples of ecosystem engineers that filter the distribution of soil fauna in many different environments. Soils beneath plant canopy or aerial cover maintain higher moisture than the surrounding matrix soil (Coulson, Hodkinson, & Webb, 2003), low soil surface temperatures modified by plant shading (Coulson et al., 1996), and soil pH which is influenced by the amount and type of plant litter accumulated (Cornelissen, Sibma, Van Logtestijn, Broekman, & Thompson, 2011). However, studies considering macro‐invertebrate diversity in its entirety (i.e., species richness, abundance, and species assemblage composition) at abandoned cattle kraals are lacking. Therefore, the influence of soil nutrients and plant productivity on abandoned cattle kraals on macro‐invertebrate diversity in African savannas remains poorly understood. Furthermore, previous studies in African savannas mainly focused on characterization of selected taxonomically and ecologically better‐known macro‐invertebrate groups (such as Isoptera: termites, Coleoptera: beetles, Megadrilacea: earthworms, and Hymenoptera: ants) and identification limited to higher taxa mainly due to the lack of taxonomic capacity (Ayuke et al., 2009; Dangerfield, 1997; Warui, Villet, & Young, 2004).

Therefore, this study aimed to test the effect of herbaceous productivity and soil nutrients as filters of macro‐invertebrate assemblages two decades after kraal abandonment in a semi‐arid African savanna. Specifically, the objectives of the study were to (a) compare soil nutrient properties and plant productivity variables (herbaceous biomass and aerial and basal covers) between abandoned cattle kraals and the surrounding savanna matrix, (b) compare belowground and aboveground macro‐invertebrate assemblages (richness, abundance, and ultimately diversity) between abandoned cattle kraals and surrounding savanna matrix, and (c) determine the relationship between aboveground and belowground macro‐invertebrate species with herbaceous productivity variables and soil nutrients, respectively. We hypothesized that soil in abandoned cattle kraals is more nutrient‐rich, because of the accumulated dung and the positive feedback loop maintained by browsing and grazing animals which drop their dung and urine during foraging (Augustine, 2003; Augustine et al., 2003; Chikorowondo et al., 2018; van der Waal et al., 2011). Secondly, we expected herbaceous productivity to be greater on abandoned cattle kraals than the surrounding savannas, because of the higher soil nutrients (Chikorowondo et al., 2018; van der Waal et al., 2011), and lastly, we expected higher macro‐invertebrate diversity on cattle kraals due to elevated soil nutrients and because of the extreme semi‐arid conditions at our study site, soil macro‐invertebrates are likely to persist where there is higher plant biomass production (Rusek, 2000). Our study adds a new dimension on the contribution of abandoned cattle kraals to the diversity of both belowground and aboveground macro‐invertebrates in African savannas and elsewhere in the world.

## 2 MATERIALS AND METHODS

### 2.1 Study area

The study was conducted in the Save Valley Conservancy, southeastern lowveld of Zimbabwe (20°05′S, 32°00′E), and it covers an area of about 3,387 km^2^. Previously, the area was used for cattle ranching until 1992 when it shifted to wildlife conservation (Waterhouse, 1994). The area lies in the semi‐arid savanna zone with a mean daily temperature of 35°C and rainfall ranging between 300 and 500 mm per annum (Lindsey et al., 2008). The geology consists largely of gneisses and paragneisses forming gently undulating terrain with scattered kopjes. Vegetation is mainly deciduous open woodland savanna dominated by *Colophospermum mopane* woodland, *Acacia–Combretum* woodland*, Acacia tortillas* woodland, and rivulet vegetation along drainage channels and patches of granite kopje vegetation (Waterhouse, 1994). The herbaceous community is dominated by a mixture of annual and perennial grasses such as *Setaria*,* Panicum*, and *Cenchrus* species. Among the diverse wildlife species, there is African elephant (*Loxodonta africana*), African buffalo (*Syncerus caffer*), leopard (*Panthera pardus*), lion (*Panthera leo*), black rhinoceros (*Diceros bicornis*), plains zebra (*Equus quagga*), giraffe (*Giraffa camelopardalis*), impala (*Aepyceros melampus*), sable antelope (*Hippotragus niger*), and hippopotamus (*Hippopotamus amphibius*).

### 2.2 Experimental design

In order to identify the location of the former cattle kraals, we engaged a former cattle herder who worked at the conservancy during the era of cattle ranching. The exact location of the abandoned kraals was confirmed using vegetation and remnant features such as management roads, water troughs, and fencing posts. The kraals were separated by at least 4 km from each other. Fifteen abandoned kraals were identified, and 12 (with a standard measurement of 100 m × 100 m) were sampled. Similar‐sized control plots were marked 200 m from each sampled kraal midpoint, at the same topographical level in order to minimize the effect of topography, soil variations, and the piosphere effect (Augustine, 2003; Augustine et al., 2003; Porensky, Bucher, Veblen, Treydte, & Young, 2013; van der Waal et al., 2011). All sampling was conducted in the wet season (November 2014 to April 2015) when vegetation is best represented (Walker, 1976) and a period of high activity of macro‐invertebrates (Alexander et al., 1993).

### 2.3 Soil sampling and analyses

To establish each random soil collection point, two random numbers between 0 and 100 were generated from a scientific calculator and the first one used as the *x*‐coordinate and the second one as the *y*‐coordinate. Using two sides of the plot as cartesian plane *x*‐ and *y*‐axes, five points were located and soil samples collected using a soil auger (with a diameter of 8.2 cm) to a depth of 15 cm and thoroughly mixed to obtain a composite sample. In order to ascertain that kraal sites are nutrient‐rich patches, soil samples were analyzed for available phosphorus (P), mineral nitrogen (N), extractable potassium (K), exchangeable calcium (Ca), sodium (Na), magnesium (Mg), organic carbon (C), and pH. Analyses were based on the standard laboratory techniques for soil analyses (Alexander et al., 1993; Motsara & Roy, 2008). Mineral N was determined by micro‐Kjeldahl digestion followed by distillation, and available P was measured spectrophotometrically following the Olsen method and extractable K by a flame photometry (Motsara & Roy, 2008). Exchangeable cations (Ca, Mg, and Na) were extracted with ammonium acetate and then measured using atomic absorption spectrophotometry (Alexander et al., 1993). Soil pH was measured using water and CaCl_2_ suspension, and organic C was determined volumetrically following procedures described by Motsara and Roy (2008). All laboratory soil analyses were conducted at the Chemistry and Soil Research Institute of the Government of Zimbabwe's Ministry of Agriculture, Mechanisation and Irrigation Development.

### 2.4 Vegetation sampling

Nine transects 100 m long (10 m apart) were marked at each abandoned kraal and its control plot in order to assess herbaceous vegetation. Herbaceous basal and aerial covers were estimated in 11 (1 m^2^) quadrats that were marked 10 m apart along the marked transects. Basal and aerial cover estimations followed the 8‐point scale by Walker (1976), with quadrats with no plants having a score of 0, and those with plants were scored: 1 = 1%–10% coverage, 2 = 11%–25% coverage, 3 = 26%–50% coverage, 4 = 51%–75% coverage, 5 = 76%–90% coverage, 6 = 91%–99% coverage, and 7 = 100% coverage.

Herbaceous biomass was estimated for each sampling plot by measuring the compressed height with a disk pasture meter dropped twice in every quadrat from the top most part of the central rod (the stopper) (Trollope, 1990). Biomass was then calculated using the same equation of Trollope (1990) as the vegetation is similar as follows:

Biomass(kg/ha)=(√X×2,260)−3,019

where X is the average disk height (cm) for the sampling plot.

### 2.5 Macro‐invertebrate sampling

The belowground macro‐invertebrates were sampled using soil monoliths and Berlese funnels. Ten soil monoliths (0.25 m × 0.25 m × 0.2 m) were taken randomly from each sampling plot using a steel monolith. The soil monoliths were put in closed buckets and transported back to the station for sorting in a closed room to prevent escape of macro‐invertebrates. The soil monoliths were removed and sifted carefully by hand on cardboard trays picking visible macro‐invertebrates, and the remaining macro‐invertebrates were flushed out using Berlese funnels (Boyce, 2005).

Aboveground macro‐invertebrates were sampled only when the wind was calm and on sunny days between 7 a.m. and 11 a.m. The order in which cattle kraals and savanna control plots were visited for sampling was randomized. To capture aboveground crawling macro‐invertebrates, five sampling grid locations were established at each kraal: one at the center of the kraal and the other four placed one in each corner of the kraal. A similar sampling protocol was used for the nearby savanna control plots. The 10 pitfall traps were arranged in a 5 × 2 grid, separated by 5 m from each other at every sampling location. Each pitfall trap made of polyethylene container (13 cm in height and 5.5 cm in diameter) was buried to rim level and filled with odorless soapy water made from OK Zimbabwe dishwashing liquid to avoid escape of captured macro‐invertebrates (Sørensen, Coddington, & Scharff, 2002; Warui et al., 2004). Contents were collected once after 24 hrs and placed in 70% alcohol prior to identification. Sweep nets (40 cm diameter) were swung randomly along strips (10 m wide) by two sweepers, each covering a width of 5 m to capture both flying and resting insects. Contents of the nets were emptied into glass bottles after every 10 sweeps. Sweeping was only made once at each sampling plot. Sweeping time was standardized to 25 min per 100 m × 10 m strip, excluding time that was spent emptying the nets into glass bottles. This exercise was carried out on different days after monolith extraction and pitfall collections.

Sorting and identification of specimens were performed by morphologically classifying macro‐invertebrates to the lowest taxonomic level possible (order, family, genus, and species) using field guide to insects of southern Africa (Picker, Griffiths, & Weaving, 2004), museum specimens, photographs, and identification keys (e.g., Isoptera (Mitchell, 1980; Uys, 2002), Lepidoptera (Pinhey, 1977), Coleoptera (Pinhey, 1975)). Specimens were identified at the Bulawayo Natural History Museum, and voucher specimens are housed at Bindura University of Science Education.

### 2.6 Data analyses

Data were tested for normality and homogeneity of variance and transformed when failed to meet the assumptions of normality. All percentage data were arcsine‐transformed before analysis. All univariate summary statistics were calculated in Past v.3 (Hammer, 2001) unless otherwise stated. Analyses for belowground and aboveground macro‐invertebrates were conducted separately. Diversity of macro‐invertebrates was assessed using the observed species richness and abundance, evenness, and diversity indices (a) Shannon–Wiener (H′) and (b) Hill number (N1). Compositional differences in macro‐invertebrate assemblages and patterns in the data between abandoned kraals and control plots were explored using the analysis of similarity (ANOSIM) in R software (R Development Core Team 2011). We used a constrained redundancy analysis (RDA) in CANOCO 4.5 (Lepš & Šmilauer, 1999) to test the relationship between aboveground and belowground macro‐invertebrates with herbaceous productivity and soil nutrients, respectively. The RDA was selected because the first axis of the detrended correspondence analysis of the data set had a gradient length <4 standard deviation units (Leps & Smilauer, 2003). Immature arthropods that could not be classified were eliminated from the analysis (Riggins, Davis, & Hoback, 2009). Abundances were log_10_‐transformed in order to weigh common and rare species more equally for a RDA (Lepš & Šmilauer, 1999). Significance of axes and explanatory variables was tested by Monte Carlo tests (999 permutations), and ordination diagrams were constructed for the first and second axes. Comparisons of soil nutrient concentration, herbaceous productivity (biomass and aerial and basal covers), and diversity measures between abandoned kraals and control plots were compared using paired *t*‐test. Sampling adequacy of macro‐invertebrates was assessed using individual‐based rarefaction curves computed in Estimate S (Colwell, 2000).

## 3 RESULTS

### 3.1 Soil characteristics and herbaceous productivity on and off abandoned kraals

**Figure 1:**
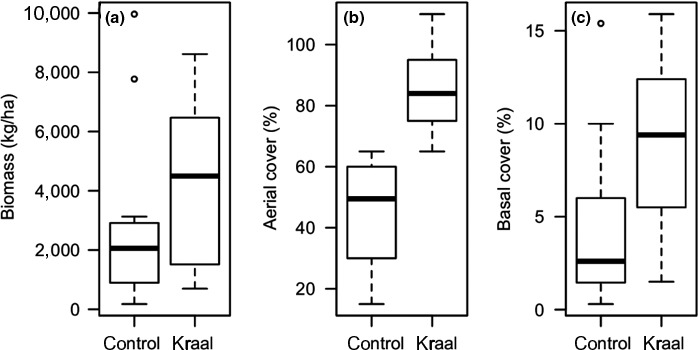
Comparisons of herbaceous productivity variables, biomass (a), aerial cover (b), and basal cover (c) between abandoned kraals and control plots in Save Valley Conservancy, Zimbabwe

Concentrations of mineral N, available P, pH, extractable Ca, and K were significantly higher (*p *<* *0.05) on abandoned kraals than on control plots (Chikorowondo, Muvengwi, Mbiba, & Gandiwa, 2017). However, no significant differences were recorded in concentrations of extractable Mg, Na, and organic C between abandoned kraals and control plots (Chikorowondo et al., 2017). Herbaceous productivity variables were significantly influenced by location (control plots vs. abandoned kraals). Herbaceous aerial cover, basal cover, and biomass were significantly higher on kraals than controls plots (all *p *<* *0.05, Figure 1).

### 3.2 Macro‐invertebrate community composition

A total of 1,852 individual soil macro‐invertebrates were collected during the study period. Aboveground macro‐invertebrates accounted for 1,289 individuals with 61% found on abandoned kraals. Abandoned kraals were dominated by family Formicidae: *Pachycondyla tarsata* (25%) and *Myrmicaria natalensis* (21%) and Lygaeidae: *Carbula marginella* (7%). Control plots were also dominated by *P. tarsata* (20%), *M. natalensis* (12%), and Pyrrhocoridae*: Dysdercus intermedus* (12%).

Belowground macro‐invertebrates had 563 individuals, with 64% occurring on abandoned kraals. Dominant invertebrates found on abandoned kraals were Isoptera: *Macrotermes michaelseni* (22%), Tenebrionidae: *Tenebrio molitor* (23%), and Pyrrhocoridae sp. (19%). Control plots had 201 individuals dominated by *M. michaelseni* (28%), *D. intermedus* (17%), and Tenebrionidae: *Zophosis mniszechi* (11%) (see Appendix 1).

**Figure 2:**
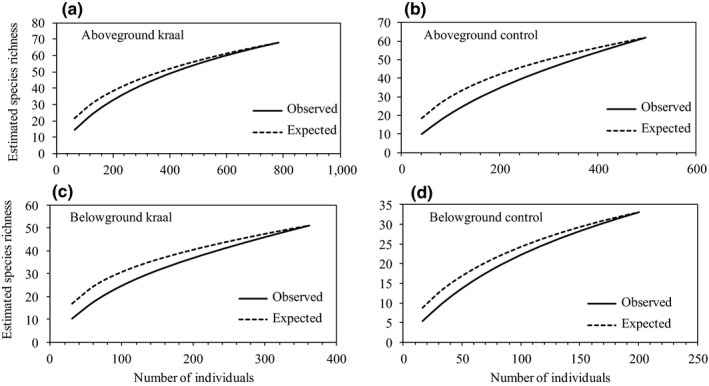
Individual‐based macro‐invertebrate species accumulation curves for aboveground kraal sites (a), aboveground control sites (b), belowground kraal sites (c), and belowground control sites (d) in Save Valley Conservancy, Zimbabwe

Species accumulation curves for aboveground macro‐invertebrates were steeper for controls compared with kraals which indicate that for the same number of individuals captured, more species were recorded for control plots (Figure 2). For belowground macro‐invertebrates, the accumulation curves for both observed and expected number of species were steeper for kraal sites compared with controls (Figure 2). All curves started to level off with an increase in the number of invertebrate individuals, but none reached an asymptote (Figure 2). Similarly, ANOSIM revealed no significant differences between kraals and control plots for macro‐invertebrate assemblages, either aboveground (global *R*_ANOSIM_ = −0.019, *p* = 0.605) or belowground (global *R*_ANOSIM_ = 0.12, *p* = 0.144).

### 3.3 Macro‐invertebrate diversity on and off abandoned kraals

Species richness was significantly higher on kraals than on control plots for both the aboveground and belowground macro‐invertebrates (*p* =* *0.01, *p* = 0.002, respectively, Table 1). Abundance of belowground macro‐invertebrates was significantly higher (*p *=* *0.004) on kraals than on controls. Although the abundance of aboveground macro‐invertebrates was greater on abandoned kraals than on controls, this difference was not significant (*p *=* *0.111). Diversity (Shannon–Wiener H′ and Hill N1) was significantly higher (both *p *=* *0.01) on abandoned kraals for belowground macro‐invertebrates. However, no significant differences (both *p *>* *0.1) in diversity were recorded for aboveground macro‐invertebrates (Table 1).

**Table 1:** Diversity comparisons between abandoned kraals and control plots for aboveground and belowground macro‐invertebrates (mean ± standard error)

	Kraal	Control	Significance
Aboveground			
Species richness	15.83 ± 2.59	9.92 ± 1.74	*t*_11_ = 4.24; *p *=* * **0.010**
Abundance	66.92 ± 13.96	41.42 ± 10.64	*t*_11_ = 1.74; *p *=* *0.110
Evenness	0.54 ± 0.07	0.65 ± 0.07	*t*_11_ = −1.34; *p *=* *0.208
Shannon H′	1.89 ± 0.15	1.62 ± 0.15	*t*_11_ = 1.46; *p *=* *0.172
Hill N1	7.57 ± 1.25	5.76 ± 0.95	*t*_11_ = 1.54; *p *=* *0.151
Belowground			
Species richness	10.33 ± 1.20	5.42 ± 0.67	*t*_11_ = 4.01; *p *=* * **0.002**
Abundance	30.17 ± 4.10	16.75 ± 3.05	*t*_11_ = 3.58; *p *=* * **0.004**
Evenness	0.70 ± 0.03	0.76 ± 0.07	*t*_11_ = −0.91; *p *=* *0.380
Shannon H′	1.91 ± 0.10	1.25 ± 0.40	*t*_11_ = 4.25; *p *=* * **0.001**
Hill N1	7.18 ± 0.84	3.91 ± 0.60	*t*_11_ = 3.36; *p *=*** *** **0.006**

Note

Significant *p*‐values (*p *<* *0.05) are shown in bold.

### 3.4 The relationship between macro‐ invertebrate community and environmental variables

#### 3.4.1 Aboveground macro‐invertebrates

**Figure 3:**
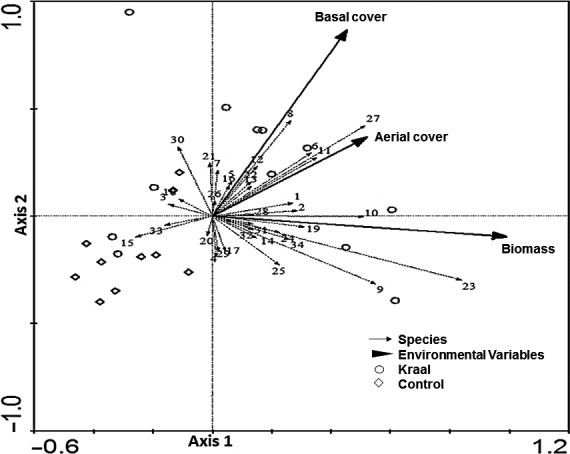
Ordination biplot of the redundancy analysis (RDA) for the aboveground macro‐invertebrate species and herbaceous plant variables (plant biomass and aerial and basal covers) from abandoned kraals and control plots in Save Valley Conservancy. 1:*Acraea* sp.; 2: *Acrididae* juvenile; 3: *Adesmia schouteni*; 4: *Altractonotus mulsanti*; 5: *Anachalcos convexus*; 6: *Aspilocoryphus fasciativentris*; 7: *Belenois aurota*; 8: *Carbula marginella*; 9: *Catacops melanostica*; 10: *Cletus* sp.; 11: *Copris elpharon*; 12: *Cubionidae* sp.; 13: *Dieuches armipes*; 14: *Dysdercus intermedius*; 15: *Geocnethus plagiata*; 16: *Gnaphosidae* larvae; 17: *Lepidoptera* larvae; 18: *Ligariella* sp.; 19: *Lycosidae* juvenile; 20: *Messor capensis*; 21: *Morphacris fasciata*; 22: *Mylabris pollita*; 23: *Myrmicaria natalensis*; 24: *Oedaleus* sp.; 25: *Oncopeltus famelicus*; 26: *Oxycarenus hyalipennis*; 27: *Pachycondyla tarsata*; 28: *Phaneratoma arnoldii*; 29: *Piezomastax* sp.; 30: *Plagiodera caffra*; 31: *Ruspolia differens*; 32: *Scantius forsteri*; 33: *Tenebrio molitor*; and 34: *Zophosis mniszechi*

Ordination RDA results showed that the first three axes explained 26.6% of the cumulative variance of species data (Table 2). Herbaceous biomass and aerial cover were positively correlated with axis 1. Monte Carlo test results showed that both the first axis (*F* = 4.192, *p *=* *0.001) and all axes combined (*F* = 2.149, *p *=* *0.001) explain significant variation in macro‐invertebrate data and the species–environment correlation for all the three axes was above 0.68. The first axis had a strong species–environment correlation (0.881) and represents a gradient of increasing herbaceous biomass (Spearman's *r *=* *0.995, *p *=* *0.00012). Herbaceous biomass had a strong association with *M. natalensis* and *Cletus* sp. (with the highest positive scores with axis 1), whereas the second axis represented a gradient of increasing herbaceous basal cover (Spearman's *r *=* *0.868, *p *=* *0.0023) and the highest positive scores with *C. marginella* and *P. tarsata* (Figure 3).

**Table 2:** Ordination RDA results for aboveground and belowground soil invertebrates and environmental variables for the first three and four axes, respectively, showing the relationship between the macro‐invertebrate community and the environmental variables on abandoned kraals and control plots in Save Valley Conservancy

	Axis 1	Axis 2	Axis 3	Axis 4
Aboveground macro‐invertebrates				
Eigenvalues	0.173	0.064	0.029	
Species–environment correlations	0.881	0.700	0.684	
Cumulative % variance of species data	17.3	23.7	26.6	
Cumulative % variance of species–environment relation	65.1	89.1	100	
Intraset correlation coefficients				
Basal cover	0.454	0.868	0.202	
Biomass	0.995	−0.097	0.026	
Aerial cover	0.521	0.368	−0.770	
Belowground macro‐invertebrates				
Eigenvalues	0.063	0.051	0.032	0.028
Species–environment correlations	0.8	0.683	0.663	0.523
Cumulative % variance of species data	6.3	11.5	14.7	17.5
Cumulative % variance of species–environment relation	34.4	62.2	79.7	94.9
Intraset correlation coefficients				
Mineral N	0.320	0.054	0.796	−0.481
Available P	0.746	0.149	0.140	0.470
pH	0.246	−0.040	0.510	0.235
Extractable K	0.050	0.694	0.678	0.148
Organic C	0.578	0.433	−0.503	−0.301

#### 3.4.2 Belowground macro‐invertebrates

**Figure 4:**
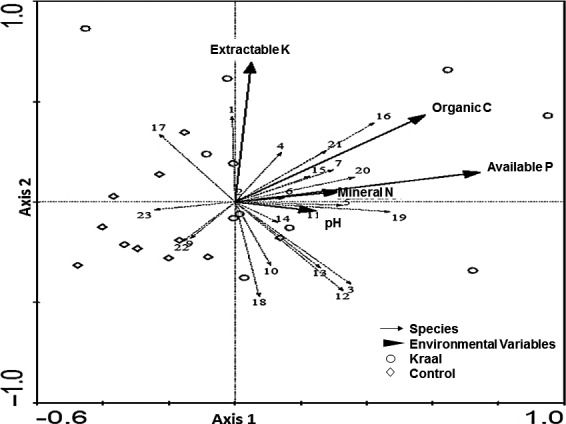
Ordination biplot of the redundancy analysis (RDA) for the belowground macro‐invertebrate species and herbaceous soil variables (extractable K, organic C, available P, mineral N, and pH) from abandoned kraals and control plots in Save Valley Conservancy. 1: *Blaberidae* sp.; 2: *Blattidae* larvae; 3: *Camponotus maculatus;* 4: *Carabidae* larvae; 5: *Carbula marginella*; 6: *Cormocephalus* sp.; 7: *Dysdercus intermedius*; 8: *Formicidae* larvae; 9: *Geocnethus plagiata*; 10: *Gnaphosidae* larvae; 11: *Hodotermes mossambicus*; 12: *Lepidoptera* larvae; 13: *Lycosidae* larvae; 14: *Macrotermes michaelseni*; 15: *Pachycondyla tarsata*; 16: *Pachylister caffer*; 17: *Parena ferruginea*; 18: *Scantius forsteri*; 19: *Schizonycha valida;* 20: *Scolopendra morsitans*; 21: *Tenebrio molitor*; 22: *Tetraponera* sp.; and 23: *Zophosis mniszechi*

Ordination RDA results showed that the first four axes explained 17.5% of the cumulative variance of species data (Table 2). The cumulative species–environment correlation of the first four axes was 94.9%. All the axes had species–environment correlations >0.50. Organic C, P, and N were positively correlated with axis 1. The first axis had a strong species–environment correlation (0.80) and represents a gradient of increasing available P (*r *=* *0.75, *p *=* *0.002) and organic C (*r *=* *0.58, *p *=* *0.034). Available P and organic C had an association with *Pachylister caffer* and *Scolopendra morsitans* (with the highest positive scores with axis 1). The second axis seems to represent a gradient of increasing extractable K (*r *=* *0.69, *p *=* *0.003) and the highest positive scores with *Blaberidae* sp., whereas the third axis represents mineral N (*r *=* *0.80, *p *=* *0.0012) and pH (*r *=* *0.51, *p *=* *0.031) and has an association with *Schizonycha valida* and *Hodotermes mossambicus*. *Z. mniszechi* and *Scantius forsteri* had the highest negative scores associated with axes 1 and 2 (Figure 4).

## 4 DISCUSSION

Our study provides some novel insights regarding the influence of soil nutrients and herbaceous productivity on macro‐invertebrates richness and diversity in a semi‐arid African savanna ecosystem. Higher levels of soil nutrients and herbaceous cover (productivity) recorded on abandoned kraals were in line with our hypotheses that predicted that these two variables would be higher on abandoned kraals. Macro‐invertebrate species abundance responded positively to an increase in soil nutrient and herbaceous productivity which indicates that abandoned kraals contribute to spatial heterogeneity in savanna, with potential implications on biodiversity and ecosystem functioning.

### 4.1 Influence of abandoned kraals on soil and herbaceous characteristics

We recorded higher soil nutrient concentrations on abandoned kraals than on control (savanna) plots, which concurs with findings in other studies from Kenya, Botswana, South Africa, and Kenya, respectively (Kizza et al., 2010; Muchiru, Western, & Reid, 2009; Porensky et al., 2013; van der Waal et al., 2011). Soil nutrients have been demonstrated to persist for decades after abandonment of kraals, being maintained primarily by the plant–soil–herbivore nutrient feedback loop (Augustine & McNaughton, 2006; van der Waal et al., 2011). Large herbivores are often attracted to nutritious forage growing on abandoned kraals and possibly drop dung and urine during feeding, which persistently enrich these sites. The cascading effects of soil nutrients at these sites may stimulate both soil macro‐ and micro‐invertebrates (Bardgett & Wardle, 2003), thereby accelerating nutrient mineralization. For example, soil nitrogen was higher on abandoned kraals in Kenya and South Africa than on control plots probably because of a sustained organic matter input and mineralization (Augustine, 2003; van der Waal et al., 2011). Phosphorus was also higher on abandoned kraals than on control plots due to mineral complexes that form in the soil, which minimizes leaching and external sourcing by herbivores (Augustine, 2003; Porensky & Veblen, 2012; Veblen, 2012). Soil pH was slightly acidic on both sites although control plots were more acidic than abandoned kraals probably due to the acidic leaf litter from woody species (Sayad, Hosseini, Hosseini, & Salehe‐Shooshtari, 2012). These soil results corroborate well with Veblen (2012), who recorded high levels of nitrogen and phosphorus (3.8 and 6.8 times, respectively) on abandoned kraals and an increase in base cations (Ca and Mg) on Kenyan savanna plots and further suggested the influence of site age and nutrient accumulation.

Herbaceous productivity (as measured by its correlates biomass and basal and aerial covers) was significantly higher on abandoned kraals than on control plots, likely due to the response of plants to elevated limiting soil nutrients. Other studies have demonstrated similar results that abandoned kraals harbor a higher herbaceous productivity compared to the surrounding matrix (Muchiru et al., 2009; Veblen & Young, 2010; van der Waal et al., 2011; Young et al., 2013).

### 4.2 Influence of abandoned kraal sites on macro‐invertebrate assemblages

Regardless of site (control or abandoned kraals), the observed high abundances of species belonging to orders Hymenoptera, Hemiptera, Coleoptera, and Isoptera recorded in the present study are comparable to other studies that were carried in the savannas (but not specifically focusing on abandoned cattle kraal sites: Ayuke et al., 2009; Muchane et al., 2012). Specifically, dominant families on abandoned kraals were Formicidae (ants), Scarabaeidae and Tenebrionidae (beetles), Lygaeidae (bugs), and Termitidae (termites) which also have been recorded as the dominant macro‐invertebrates on nutrient‐rich habitats elsewhere (Doblas‐Miranda, Sánchez‐Piñero, & González‐Megías, 2009; Marchão et al., 2009). The presence of Isopterans and Coleopterans in high abundances can be explained by the effect of our sampling period which coincided with their period of the highest activity.

Species richness was higher on abandoned kraals for both belowground and aboveground habitats, an indication that abandoned kraals are a favorable habitat in terms of feed quality and quantity that supports a variety of macro‐invertebrates (Wardle et al., 2004). Other studies have also recorded a high species richness of soil macro‐invertebrates on nutrient‐rich habitats (Ayuke et al., 2009; Doblas‐Miranda et al., 2009; Hemerik & Brussaard, 2002; Manyanga, Mafongoya, & Tauro, 2014). Unlike belowground macro‐invertebrates, species abundance for the aboveground habitats was similar at both sites, possibly indicating that aboveground macro‐invertebrates were less sensitive to soil nutrients and plant biomass than belowground macro‐invertebrates. In this study, a 150 m distance between abandoned kraals and control plots could have been traversed during foraging and dispersal. For example, Formicides and Isopterans forage over long distances from their nests and practice swarming when new reproductives fly to establish new colonies (Doblas‐Miranda et al., 2009). Furthermore, diversity did not differ significantly for aboveground species, contradicting our hypothesis that diversity would be higher on abandoned kraals because of higher levels of plant and litter biomass. This result is not in line with productivity–diversity hypothesis (Grime, 1973) and other empirical studies (Doblas‐Miranda et al., 2009; Laossi et al., 2008, Mathieu et al., 2009; Siemann, 1998) where macro‐invertebrate diversity significantly increased with an increase in plant productivity. This can be accounted for by the generalist feeding behavior and habitat preference (relatively unresponsive to small changes in resource quality) by aboveground invertebrates (Laossi et al., 2008).

Considering the RDA results for aboveground macro‐invertebrates, phytophagous *C. marginella*,* Cletus* sp. and predacious ants *P. tarsata* and *Mymicaria natalensis* had an association with plant biomass and aerial and basal covers. Phytophagous hemipterans could have been attracted by vigorous plants especially the dominant forb species found on abandoned kraals (Veblen, 2012), whereas predacious ants favor prey‐rich areas like beneath grasses (López, Agbogba, & Ndiaye, 2000). RDA results for belowground macro‐invertebrates indicated that the Myriapods (*S. morsitans*), Coleopterans *(Pachlister caffer*), and Isopterans (*H. mossambicus*) benefited most from high concentration of limiting soil nutrients (N, P, K, and C) on abandoned kraals. Similar findings have been recorded on nutrient‐rich areas by others (Doblas‐Miranda et al., 2009; Riggins et al., 2009; Salamon et al., 2011; Wu, Zhang, & Wang, 2015). Soils rich in N, P, K, and C are associated with high microbial biomass (Lavelle, 1997) which could have directly or indirectly attracted these species. Dung and carrion that is commonly found on abandoned kraals (Riginos et al., 2012) could have attracted a wide variety of coprophagous and necrophagous species, for instance the Coleopterans (Salamon et al., 2011). On the other hand, abandoned kraals enhance the microclimate for a variety of soil and litter dwellers through good nutrient supply for plant growth (Muchiru et al., 2009) and ultimately improved the quality and quantity of litter (Mathieu et al., 2009).

## 5 CONCLUSIONS

Our study shows that abandoned kraals as long‐term nutrient‐rich habitat patches have a significant influence on abundance and richness of macro‐invertebrates. In line with our hypothesis, abandoned kraals were still soil nutrient‐rich and had a significantly high richness and abundance of belowground macro‐invertebrates. However, the diversity of aboveground macro‐invertebrates did not respond significantly to the influence of abandoned kraals. Soil nutrient properties and herbaceous productivity had a positive influence on relative abundances of some belowground and aboveground macro‐invertebrate taxa as shown by significant correlations. Therefore, our results suggest that nutrient abundance on abandoned kraals affects the belowground macro‐invertebrates more than the highly mobile aboveground taxa. However, in order to fully ascertain the influence of habitat productivity on macro‐invertebrate assemblages, further research is needed on the horizontal mobility of each taxa and its sensitivity to resource heterogeneity at both spatial and temporal scales on nutrient hotspots in semi‐arid savannas.

## CONFLICT OF INTEREST

None declared.

## AUTHOR CONTRIBUTIONS

Justice Muvengwi, Gift Chikorowondo, Monicah Mbiba, and Edson Gandiwa designed the study. JM, GC, and MM collected and analyzed the data. JM, GC, MM, and EG discussed the results and wrote the manuscript.

## DATA ACCESSIBILITY

Data available from the Dryad Digital Repository: https://doi.org/10.5061/dryad.fk81jf2

## Appendix 1 Checklist of macro‐invertebrates found on abandoned kraals and control plots in Save Valley Conservancy, Zimbabwe

### 1.1

**Table:**

Order	Family	Genus/ species	Total	
			Kraals	Controls
*Amblypygi*	*Amblypygidae*	*Damon* sp.	2	–
*Acarina*	*?*	***	–	1
*Araneae*	*Araneidae*	***	2	–
	*Corrinnidae*	***	4	–
	*Cubionidae*	***	8	–
	*Gnaphosidae*	***	8	7
	*Heteropodidae*	***	–	1
	*Lycosidae*	***	19	4
	*Salticidae*	***	3	–
	*Selenopidae*	*Selenopes* sp.	2	–
	*Thomisidae*	***	1	2
	*Zodarridae*	***	1	1
*Blattodea*	*Blaberidae*	*Hostilia* sp.	13	5
	*Blattidae*	Juvenile	5	–
*Coleoptera*	*Carabidae*	Larva	15	2
		*Cypholoba alveolata*	1	–
		*Anthia pachyoma*	2	–
		*Altractonotus mulsanti*	2	5
		*Poecilothais tetragramma*	1	–
		*Dromica spectabilis*	–	2
		*Parena ferruginea*	2	7
	*Chysomelidae*	*Plagiodera caffra*	3	6
		*Crabonites equestris*	1	2
	*Coccinellidae*	*Cheilomenes lunata*	2	–
		*Henosepilachna bifasciata*	1	–
*Curculionidae*	*Calodemas nickerli*	–	2
*Histeridae*	*Pachylister caffer*	8	–
*Meloidae*	*Mylabris pollita*	3	2
*Scarabaeidae*	*Anachalcos convexus*	8	12
	*Aphodius* spp.	1	1
	*Copris elpharon*	19	2
	Larva	1	1
	*Kheper nigroaeneus*	–	1
	*Onitis fulgidus*	1	–
	*Onitis westermanni*	2	–
	*Schizonycha valida*	11	2
Tenebrionidae	*Adesmia schouteni*	31	22
	*Anomalipus polymorphus*	–	2
	*Endustomus parallelogrammus*	1	–
	*Eurychora* sp.	4	–
	*Phanerotoma arnoldi*	4	6
	*Phanerotoma scobicolle*	–	1
	*Psammodes virago*	4	–
	*Stips dohrni*	1	–
	*Tenebrio molitor*	50	7
	*Zophosis mniszechi*	31	32
Trogidae	*Trox sulcatus*	1	–
Diptera	Asilidae	*Philodicus* sp.	2	–
Hemiptera	Coreidae	*Cletus* sp.	17	2
		*Oxycarenus hyalipennis*	16	1
		*Pephricus* sp.	1	1
	Cydnidae	*Geocnethus plagiata*	8	8
	Lygaeidae	*Aspilocoryphus fasciativentris*	41	5
		*Carbula marginella*	60	5
		*Dieuches armipes*	1	6
		*Graptostethus servus*	1	–
		***	3	2
		*Oncopeltus famelicus*	25	22
	Pentatomidae	*Nezara virudia*	1	1
		***	5	–
		*Diploxys fallax*	–	1
	Pyrrhocoridae	*Dysdercus intermedus*	71	97
		*Scantius forsteri*	28	11
	Reduviidae	*Leptodena acanthoocephalum*	1	–
Homoptera	Eurybrachidae	*Aselgela ramulifera*	3	–
Hymenoptera	Formicidae	*Camponotus maculatus*	17	5
		*Messor capensis*	7	25
		*Myrmicaria natalensis*	161	68
		*Pachycondyla tarsata*	275	75
		***	6	–
		*Polyrhachis* sp.	–	1
		*Tetraponera* sp.	4	4
	Mutillidae	*Mutilla astarte*	2	–
	Ichneumonidae	*Archibracon serviellei*	1	–
Isoptera	Hodotermitidae	*Hodotermes mossambicus*	19	8
	Termitidae	*Macrotermes michaelseni*	26	71
Lepidoptera	Nymphalidae	*Acraea* sp.	16	7
		*Charaxes* sp.	2	2
	Pieridae	*Belenois aurota*	2	7
	?	Larva	20	7
Mantodea	Mantidae	Juvenile	2	–
		*Ligariella* sp.	3	2
		***	2	1
		*Tarachodes*	–	1
	Thespidae	*Hoplocoryphella grandis*	1	–
Myriapoda	Odontopygidae	***	4	–
	Scolopendromopha	*Cormocephalus* sp.	5	6
		*Scolopendra morsitans*	8	2
Neuroptera	Myrmeleontidae	*Palpares* sp. (larva)	2	1
Orthoptera	Acrididae	*Acrida acuminata*	4	–
		*Catacops melanostica*	5	5
		*Humbe tenuicornis*	3	–
		Larva	1	3
		*Morphacris fasciata*	15	2
		*Oedaleus* sp.	6	9
		***	1	–
		*Orthoctha* sp.	–	4
		*Piezomastax* sp.	21	6
	Gryllidae	*Juvenile*	6	2
		***	1	–
	Lentulidae	*Mecostibia mopane*	1	–
	Pamphagidae	*Lamarkiana puntosa*	–	1
		***	3	–
	Tettigoniidae	*Russipolia differens*	7	1
Scorpiones	Scorpionidae	*Opistophthalmus glabrifrons*	2	–
	Buthidae	*Parabothus transvaalicus*	–	1
Solpugida	Solfugidae	***	–	1

Note

Symbols: ?: unknown family; *: unknown genus/species.
